# Mycobacterial Tyrosine Phosphatase PtpB Affects Host Cytokine Expression by Dephosphorylating ERK1/2 and STAT3

**DOI:** 10.1016/j.mcpro.2025.101067

**Published:** 2025-09-09

**Authors:** Tianxian Liu, Yameng Fan, Yijia Chen, Shuyu Xie, Jun-Yu Xu, Minjia Tan, Bang-Ce Ye

**Affiliations:** 1 Laboratory of Biosystems and Microanalysis, State Key Laboratory of Bioreactor Engineering, East China University of Science and Technology, Shanghai, China; 2 State Key Laboratory of Drug Research, Shanghai Institute of Materia Medica, Chinese Academy of Sciences, Shanghai, China; 3 Zhongshan Institute for Drug Discovery, Shanghai Institute of Materia Medica, Chinese Academy of Sciences, Zhongshan, China

**Keywords:** mycobacteria, tyrosine phosphatases PtpB, tyrosine phosphoproteomic, ERK1/2, immune response

## Abstract

*Mycobacterium tuberculosis* (Mtb) tyrosine phosphatases PtpA and PtpB have been widely reported to affect host immunity response and bacterial intercellular survival. However, a comprehensive investigation into the impact of PtpA and PtpB on host phosphorylation, specifically in their roles as tyrosine phosphatases, has not yet been reported. In this study, we first conducted the potential dephosphorylation substrates map of PtpA and PtpB within the host. Our findings demonstrated that PtpB significantly decreased the phosphorylation levels of ERK1/2 and STAT3. Subsequent analysis indicated that PtpB modulated the production of cytokine TNF and IL-1β by dephosphorylating ERK1/2 and preventing its nuclear translocation. PtpB also reduced IL-6 and IL-1β expression by dephosphorylating STAT3. The *in vivo* experiment demonstrated increased bacterial survival and reduced cytokine expression in the PtpB-overexpression strain. Consequently, our findings demonstrate that Mtb tyrosine phosphatases PtpA and PtpB play critical roles in the global tyrosine phosphorylation landscape within the host. Specifically, PtpB modulates cytokine expression through the dephosphorylation of ERK1/2 and STAT3.

## Highlights

• First comprehensive characterization of the substrate landscape of *Mycobacterium* phosphatases PtpA and PtpB in host cells.
• PtpB inhibits nuclear translocation of ERK1/2 *via* dephosphorylation, thereby suppressing host cytokine expression.
• PtpB reduces IL-6 expression by disrupting host JAK/STAT signaling pathways.

## Graphical Abstract

**Figure:**
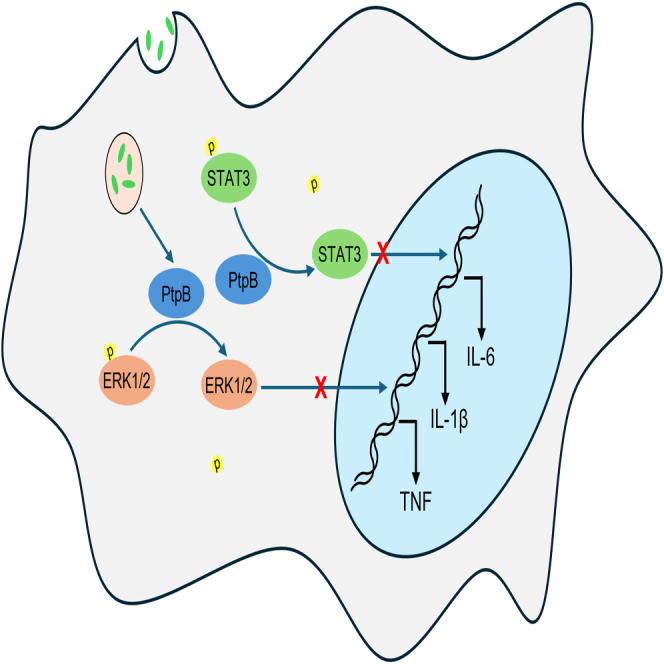

## In Brief

This study comprehensively characterizes the substrate landscape of *Mycobacterium* tyrosine phosphatases PtpA and PtpB within host systems. We demonstrate that PtpB suppresses ERK1/2-mediated cytokine expression by reducing ERK1/2 phosphorylation and inhibiting its nuclear translocation. Furthermore, PtpB downregulates STAT3 phosphorylation, thereby impairing JAK-STAT signaling and subsequent IL-6 cytokine secretion. These findings were validated in both cellular and animal models.

*Mycobacterium tuberculosis* (Mtb) is a pathogenic bacterium that causes tuberculosis (TB) seriously threatens human health. According to reports from the World Health Organization (WHO) in 2023, TB remains a major concern, resulting in approximately 1.3 million deaths annually (1). Mtb is an adaptable intracellular pathogen that evades the host immune response through various strategies, facilitating the establishment of the chronic infection. Numerous effector proteins have been identified, elucidating their roles in facilitating Mtb evasion of immune-mediated damage during infection (2, 3, 4, 5, 6).

Mtb kinase and phosphatase system comprises an extensive family that includes 11 serine/threonine protein kinases (PknA to PknL), a single serine/threonine protein phosphatase (Pstp), two tyrosine phosphatases (PtpA and PtpB), and a single tyrosine kinase (PtkA) (7, 8, 9, 10, 11). Some of these proteins are secreted into host and then disturb the normal signaling pathways during Mtb infection. For instance, PknG has been widely reported to influence the fusion of phagosomes and lysosomes in macrophages, as well as to modulate host autophagy, thereby facilitating the intracellular survival of bacteria (2, 12). PtpA and PtpB are essential virulence factors in Mtb infection. PtpA has been reported to facilitate ferroptosis *via* the PtpA-PRMT6-H3R2me2a pathway and to regulate host gene expression (13). PtpA has been also reported to influence immune responses by manipulating the host ubiquitin system (14, 15, 16). PtpB, a tyrosine phosphatase exhibiting phosphoinositide phosphatase activity (17, 18), has been reported to inhibit host pyroptosis and cytokine release through the dephosphorylation of phosphatidylinositol. Moreover, PtpB modulates cytokine expression *via* the NLRP3 inflammasome pathway (4). NLRP3 is a multiprotein cytoplasmic complex that functions as a cytosolic immune sensor. This inflammasome activates caspase-1, leading to gasdermin D (GSDMD) cleavage and subsequent membrane pore formation. However, PtpB inhibits cytokine release by blocking GSDMD translocation to the membrane. Notably, Chai *et al.* observed that PtpB-mediated suppression of TNF and IL-6 occurs independently of NLRP3, though the precise mechanism linking PtpB to TNF/IL-6 regulation remains uncharacterized (4). Both PtpA and PtpB have been reported to dephosphorylate key MAPK proteins including p38, JNK, and ERK (19, 20, 21). However, the dephosphorylation status of each substrate in these studies was individually validated by Western blot, and a comprehensive investigation of the impact of Mtb tyrosine phosphatase PtpA and PtpB on alterations of global tyrosine phosphorylation within the host is yet to be undertaken. Furthermore, the causal relationship between PtpA/PtpB-mediated alterations in protein phosphorylation and cytokine expression remains incompletely characterized, as systematic functional validation of this axis is currently lacking.

In this study, we initially identified the global potential tyrosine phosphorylation substrates of PtpA and PtpB utilizing SH2-CNBR superbinder technology as previously reported (22, 23). Our findings indicated that PtpB significantly downregulates ERK1/2 phosphorylation levels. Subsequent experiments revealed that PtpB directly interacts with ERK1/2, thereby inhibiting its activity, nuclear translocation, and the expression of cytokines. And those studies. Furthermore, PtpB reduced STAT3 phosphorylation and then decreased IL-6 cytokine expression. We confirmed that PtpB dephosphorylates ERK1/2 and STAT3, thereby inhibiting cytokine expression at the level of infection and promoting mycobacteria survival. Our study uncovered the function of PtpB as a tyrosine phosphatase in the regulation of host cytokine responses, which underscores the importance of this mechanism in regulating host signaling pathways.

## Experimental Procedures

### Cell Culture and Bacterial Stains

293T and Raw264.7 cells were cultured in DMEM medium supplemented with 10% fetal bovine serum (FBS). Both cell lines were cultured with 100 U/ml Penicillin and 100 mg/ml Streptomycin and incubated at 37 °C with 5% CO_2_. For Raw264.7, cells were plated into 6-well plate overnight and treated with 10 μg/ml U0126 or SCH772984 for 4 h and following added muramyl dipeptide (MDP) at the final concentration of 10 μM for another 12 h or 200 ng/ml LPS stimulated for 6 h. *Mycobacterium smegmatis* MC2 155 (*M. smeg*) was incubated in lysogeny broth (LB) medium at 37 °C.

### Reagents

In this study, we used: Anti-phosphotyrosine antibody (ZMS16282, Sigma Aldrich), anti-Flag (A8592, Sigma Aldrich), HRP-conjugated β-Actin rabbit mAb (AC028, Abclonal), liposomal transfection reagent (40802ES03, Yeasen), polybrene (40804ES76, Yeasen), trypsin (HLSTRY001 C, Hualishi Scientific), Filgotinib (HY-18300, MCE), U0126 (HY-12031A, MCE), SCH772984 (HY-50846, MCE), MDP (HY-127090, MCE), LPS (HY-D1056, MCE), anti-Flag M2 (Sigma-Aldrich, A2220), JAK1 rabbit mAb (A25841, Abclonal), Stat3 rabbit mAb (4904S, Cell Signaling Technology), Phospho-Stat3 rabbit mAb (9145S, Cell Signaling Technology), Stat5 rabbit mAb (94205S, Cell Signaling Technology), Phospho-Stat5 rabbit mAb (9359S, Cell Signaling Technology), ERK1/2 rabbit mAb (A4782, Abclonal), Phospho-ERK1/2 rabbit mAb (AP0974, Abclonal), Rabbit mAb Alexa Fluor 488 labelled secondary antibody (A27034, Thermo Fisher), Alexa Fluor 594 labelled secondary antibody (A11004, Thermo Fisher), and Hoechst 33,342 (P0133, Beyotime).

### Experimental Design and Statistical Rationale

For proteomic and phosphoproteomic analyses, three biological replicates per group (EV, PtpA-overexpression, PtpB-overexpression) were used. Phosphorylation site intensities were normalized to their corresponding protein intensities. Data were considered significantly changed with a >1.2-fold change and a *p*-value <0.05. For qPCR validation in bacterial infection at the cell and mouse levels, at least three replicates per group were performed. Statistical significance of differences between two groups was assessed using a two-tailed unpaired Student's *t* test. Data are presented as mean ± SEM, with significance denoted as ∗*p* < 0.05, ∗∗*p* < 0.01, and ∗∗∗*p* < 0.001.

### Gene Cloning and Plasmid Construction

The template of Mtb genes PtpA and PtpB (PtpA^Mtb^, PtpB^Mtb^) was purchased from Beijing Tsingke Biotech Co., Ltd. The genes were cloned into pCDH with a 3X-Flag tag for mammalian cell transfection and cloned into PMV261 with a 6X-His tag in the N-terminal for mycobacteria transformation. The DH5а strain was used to amplify the constructed plasmids with ampicillin resistance. All constructed plasmids were verified by sequencing. Primers for plasmid construction are listed in Supplemental Table S5.

### Mammalian Cell Plasmid Transfection

293T cells were transfected with pCDH, pCDH-PtpA^Mtb,^ and pCDH-PtpB^Mtb^ using Liposomal Transfection Reagent from Yeasen Biotech Co., Ltd, according to the manufacturer’s instructions. For tyrosine phosphorylomics, cells were seeded in a 10-cm dish for 24 h before transfection, and 3 replications for each group. Cell culture medium was removed and replaced with medium without antibiotics. Furthermore, Opti-MEM (Gibco),12 μg recombined plasmid, and 30 μl Liposomal Transfection Reagent were mixed for 10 min and added to the cells, and incubated for 6 h. Cells were cultured for another 24 h before collection. For promoter transcriptional strength measurement, cells were seeded in a 24-well plate and transfected 0.5 μg pCDH-PtpA^Mtb^/PtpB^Mtb^, 0.25 μg pRL-TK, and 0.25 μg different promoter luciferin reporter plasmids per well. After compounds were treated and incubated for 24 h, cells were collected for further analysis.

### Mycobacteria Competent Cell Preparation and Plasmid Electroporation

*M. smeg* were previously activated in LB solid agar plate for 3 days and activated *M. smeg* were amplification in LB medium at 37 °C to logarithmic phase. Cells were collected and pre-clod on ice for 30 min and washed 3 times using 10% glycerin. The bacteria precipitation was resuspended in 10% glycerin and quickly freeze in liquid nitrogen. The complete bacteria were stored in −80 °C and thaw on ice for use.

The thawed *M. smeg* was mixed with PMV261-PtpA^Mtb^ or PMV261-PtpB^Mtb^ and incubated on ice for 5 min. The electroporation conditions were 3 KV and 4 ms. After electroporation, *M. smeg* were incubated in LB medium for 3 h at 37 °C, and all the bacteria were steaked onto the LB agar plate with kanamycin and cultured for 3 days at 37 °C.

### Protein Extraction and Digestion

For tyrosine phosphorylomics samples, 293T cells were washed twice with PBS after being collected at 600 rcf. After that, cells were resuspended in 10 ml PBS with 1 mM sodium pervanadate and incubated for 10 min at 37 °C. Cells were harvested at 600 rcf, and the precipitates were treated with lysis buffer (100 mM NH_4_HCO_3_, 8M urea, 4× phosphatase inhibitor, 2× protease inhibitor cocktail) and lysed on ice for 30 min. All cell lysates were sonicated at 300W for 3 min with 2s work and 3s break, followed by centrifugation at 21,000 g for 10 min, and the supernatant was removed to another tube and quantified by the BCA protein quantification kit.

Quantified proteins were reduced with 5 mM dithiothreitol (DTT) at 56 °C for 30 min and then alkylated with 15 mM iodoacetamide (IAA) at room temperature in the dark for 30 min. Subsequently, 500 μg proteins per sample were filtered and washed 3 times with 100 mM NH_4_HCO_3_ buffer using a 10 kDa filter every 10 min at a speed of 13,000 rcf after termination by adding 15 mM DTT for 30 min. Samples were then digested by trypsin (1:50 [w/w]) overnight at 37 °C and a second digestion with additional trypsin (1:100 [w/w]) for 4 h. All digested peptides were collected to desalt using Sep-Pak C18 columns and vacuum freeze-dried.

### TMT Labeling and High-pH HPLC Fraction

The peptide samples used for tyrosine phosphorylomics were labelled with 126-130C channels of TMT10plex. TMT reagents were dissolved in 15 μl ACN and added to peptide samples, which were dissolved in 80 μl 50 mM TEAB buffer (PH: 8.0–8.5). After reacting for 1 h at 25 °C, samples were quenched with 0.5% hydroxylamine for 15 min, and each channel peptides were mixed a little to determine labeling efficiency. Once over 95% labeling efficiency was measured, all channel samples were mixed and desalted by Sep-Pak C18 columns and vacuum freeze-dried.

The labeled peptides were fractionated by High-pH HPLC with a Waters XBridge Prep C18 column (Waters Corp). The flow rate was set to 1 ml/min, and a fraction was collected every minute. A sample of every fraction was collected, and 20 fraction samples were pooled for proteomic quantification. The remainders of the fractions were mixed to 6 fractions for further tyrosine phosphorylated peptides enrichment.

### SH2 Superbinder Preparation and Tyrosine Phosphorylated Peptides Enrichment

Triple mutant Src SH2 domain was purified from *E.coli* BL21 (DE3) as reported before (22, 23). The purified SH2 superbinder was covalently immobilized on activated CNBR Sepharose beads.

For tyrosine phosphorylation enrichment, peptide samples were dissolved in cold immunoaffinity purification (IAP) buffer (50 mM Tris-HCl, 50 mM NaCl, 10 mM Na_2_HPO_4_, pH 7.0) and incubated with SH2-CNBR superbinders overnight with gentle rotation at 4 °C. After incubation, SH2-CNBR superbinders were washed 3 times with 1 ml IAP buffer and twice with 1 ml water. Tyrosine phosphorylated peptides were eluted by 0.2% trifluoroacetic acid (TFA) and 0.2% TFA buffer with 60% ACN. Eluted peptides were vacuum freeze-dried and desalted by Sep-Pak C18 columns for further analysis.

### Coimmunoprecipitation (CoIP) Analysis

For CoIp analysis, 293T cells were transfected with pCDH or pCDH-PtpB^Mtb^ plasmids for over 24 h and then harvested. Cells were lysed by IP buffer (20 mM Tris-HCl, 0.5% NP-40, 100 mM NaCl, 0.5 mM EDTA, 2× protease inhibitor, pH 8.0) and incubated on ice for 30 min. The lysed samples were sonicated for 3 min and centrifuged for 5 min. Supernatants were removed to new tubes and incubated with 20 μl IP buffer washed mouse IgG beads at 4 °C for 4 h to remove non-specific binding. After that, IG beads were removed, and the supernatants were incubated with 20 μl anti-Flag beads at 4 °C overnight. Finally, anti-Flag beads were washed 7 times with cold IP buffer and eluted by 2× SDS loading buffer. The eluted samples were analyzed by Western blot or LC-MS/MS.

### LC-MS/MS Analysis

The process and parameters were similar to Li etc (24). A manually packed reverse-phase C18 column (C18 resin with 1.9 μm particle size; 10 cm length × 75 μm inner diameter) was inserted into a nano-HPLC system (Thermo Fisher Scientific). The samples dissolved in solvent A (0.1% formic acid and 2% ACN) were loaded at a flow rate of 300 nl/min.

For TMT labeling proteome, the analysis gradient was 70 min with solvent B (0.1% formic acid in ACN) from 2% to 47% for 64 min and 80% solvent B for 5 min. HPLC was connected to Q Exactive HF-X mass spectrometer (Thermo Fisher Scientific) with a nanospray ion source in positive mode. For MS1, survey scan m/z range 450-1650 with the mass resolution of 60,000 and the AGC targets were set to 5E5. For MS2, the ions were isolated for intensity larger than 50, 000 and multiply charged ions were fragmented by higher-energy collision dissociation (HCD) with normalized collision energy of 40%. The dynamic exclusion was set as 50 s and the resolution was 45,000. The mass range was 200 to 2000 m/z and the isolation window was 0.8 m/z.

For TMT labeling tyrosine phosphoproteome, the analysis gradient was 110 min with 2 to 30% solution B for 90 min and 80% solvent B for 5 min. Positive mode was set and survey scan m/z range 450-1650. Mass resolution 60,000 and AGC targets were set to 5E5 for MS1. The HCD, dynamic exclusion, resolution, mass range, and isolation window for MS2 were same as proteome.

For PtpB CoIP samples, the analysis gradient was 60 min with 5 to 13% solution B for 20 min, 13 to 26% solution B for 31 min, 26%-45% solution B for 5 min and 80% solution B for 3 min. HPLC was connected to Orbitrap Fusion (Thermo Fisher Scientific). For MS1, survey scan m/z range 350-1800 with the mass resolution 120,000 and the AGC targets were set to 5E5. For MS2, the detector was IonTrap with the HCD 32%. The mass range was 350-1400 m/z and isolation window was 1 m/z.

### Mass Spectrometry Data Database Search and Analysis

The raw MS data files were analyzed by Mascot (version 2.3.01) or Maxquant (version 2.0.1.0) and the database (79,038 proteins) was downloaded from Uniprot (23/11/2021). Parameters setting was listed as follows: maximum missed cleavage value was 2, enzyme was trypsin/P, the variable modifications were: acetylation of protein N-term, Oxidization(M), and the Carbamidomethy(C) was set as the fixed modification. Phosphorylation (S/T/Y) was set as variable modification for tyrosine phosphoproteome. The mass tolerance was set as 20 ppm for both precursor ions and fragment ions. The false discovery rate (FDR) was set to 1% at modification, peptide and protein levels. TMT10plex-based MS2 reporter ion quantification was chosen and phosphorylated sites localization score values lower than 0.75 were removed in our phosphoproteome data. Phosphoproteome intensity was normalized by corresponding proteome intensity, and statistical analyses were performed by two-tailed *t* test (*p* < 0.05), ratio >1.2 were considered as significantly changed sites/proteins.

### Bioinformatics Analysis

WebGestalt (http://www.webgestalt.org/, WebGestalt 2024) was used for enrichment analysis of KEGG pathway and gene ontology biology process (GO-BP). FDR was set to 0.05 and the minimum number of analytes for a category was set 5. The STRING database (version 12.0) was utilized for protein–protein interaction (PPI) analysis, and protein bcluster analysis used a confidence interaction score threshold of ≥0.4. The MODE package in Cytoscape software was used to analyze protein-protein interaction (PPI) subclusters with score ≥4.

### RNA Isolation and Quantitative Real-Time Polymerase Chain Reaction (qPCR)

Microphages or mouse tissues' total RNA were extracted with Tissue/Cell Fast RNA Extraction Kit (ABclonal) according to the manufacturer’s instructions. Isolated RNA was transcribed to complementary DNA (cDNA), and cDNA was used for qPCR analysis with 20 μl reaction mix. The data was collected by the Bio-Rad system and normalized according to *Actin*. All primers used for qPCR are listed in Supplemental Table S6.

### PtpB Purification and Peptide Dephosphorylation Assay *in vitro*

PtpB and GFP were purified from 293T overexpression cells by anti-Flag beads. The process was similar to CoIP experiments. Briefly speaking, PtpB and GFP were connected with a 3xFlag tag and overexpressed in 293T cells. Targeted proteins were enriched by anti-Flag beads and washed with IP buffer. The beads binding with PtpB or GFP and 50 ng/μl artificial phosphorylated peptide TGFLTEY(pho)VATR (Shanghai Hongtide Biotechnology) were added into the reaction buffer (100 mM Tris-HCl and 100 mM sodium acetate, pH 6). Reaction buffer was gently shaken at 37 °C for 2 h, and the peptide was desalted by ziptip and analyzed by LC-MS/MS.

### Raw264.7 Infection and Colony-Forming Unit Assay

For macrophage infection, *M. smeg* and *M. smeg*-PtpB^Mtb^ in LB medium with 0.05% Tween-80 were grown to logarithmic phase at an OD600 of 0.4 to 0.6. *M. smeg* were collected and washed once using 1xPBS, and the bacterial precipitates were supplemented with complete DMEM medium. Raw264.7 were seeded at 10,000 cells in 24-well plates per well overnight before infection. Raw264.7 were then infected with *M. smeg*/*M. smeg*-PtpB^Mtb^ with the MOI of 5 for 2 h. After that, Raw264.7 were washed twice with 1xPBS and cultured in fresh complete DMEM medium containing 25 μg/ml gentamycin for 24 h. Cells were harvested for further analysis.

### Cell Cytotoxicity Analysis

Cell activities were measured by Cell Counting Kit (CCK8). Briefly speaking, cells were seeded in a 12-well plate overnight and treated with *M. smeg*/*M. smeg*-PtpB^Mtb^. After infection, 900 μl complete DMEM medium and 100 μl CCK8 reagents were mixed and added into the plate per well and incubated at 37 °C for 30 min. Solutions were collected and centrifuged to remove precipitation, and supernatants were measured by a microplate reader at a wavelength of 450 nm.

### Immunofluorescence Microscopy

293T cells or Raw264.7 were seeded on glass-bottomed 24-well plates and transfected with plasmids or infected with bacteria. After being treated for over 24 h, the medium was removed and washed twice with 1x PBS and fixed in 4% paraformaldehyde (PFA) for 20 min at room temperature. Following, cells were washed with 1x PBS and treated with 0.5% Triton X-100 for 10 min. After three washes, cells were blocked with 3% BSA for 1 h and subsequently incubated with specific primary antibodies (anti-His: 1:800; anti-ERK1/2: 1:500; anti-Flag: 1:1000) diluted in 3% BSA at 4 °C overnight. After washing three times in 1xPBS, cells were incubated with Alexa Fluor 488 or Alexa Fluor 594-labeled secondary antibody (1:1000) for another 1 h in the dark.

Cells were washed three times and stained with 2’-(4-Ethoxyphenyl)-5-(4-methyl-1-piperazinyl)-2,5′-bi-1H-benzimidazole trihydrochloride (Hoechst 33,342) staining solution and washed 3 times with 1xPBS. Cells were imaged by a Nikon confocal laser scanning system and analyzed with ImageJ software.

### Mouse Infection

For *in vivo M. smeg* infection, bacteria were cultured to log phase and washed twice with sterile saline and diluted to an OD_600_ of 0.5. Eight- to 10-week-old BALB/c female mice were infected *via* tail intravenous injection with 0.1 ml bacterial suspension, and six mice per group. After being infected for 2 weeks, mice were sacrificed by cervical dislocation, and tissues were taken for further qPCR analysis and hematoxylin and eosin (H&E) staining.

### Ethic Statement

All animal procedures were performed in accordance with the Guidelines for Care and Use of Laboratory Animals of East China University of Science and Technology and the experiments were approved by the Animal Ethics Committee of East China University of Science and Technology with the statement number ECUST-21038.

## Results

### *M. tuberculosis* Tyrosine Phosphatases PtpA and PtpB Affect Host Receptor Tyrosine Signaling Pathways

*M. tuberculosis* (Mtb) tyrosine phosphatases PtpA and PtpB are critical virulence factors during infection. To identify potential substrates for tyrosine dephosphorylation within the host, we conducted tandem mass tag (TMT)-based quantitative proteomic and tyrosine phosphoproteomic analyses in HEK-293T cells overexpressing Mtb proteins PtpA or PtpB.

**Fig. 1:**
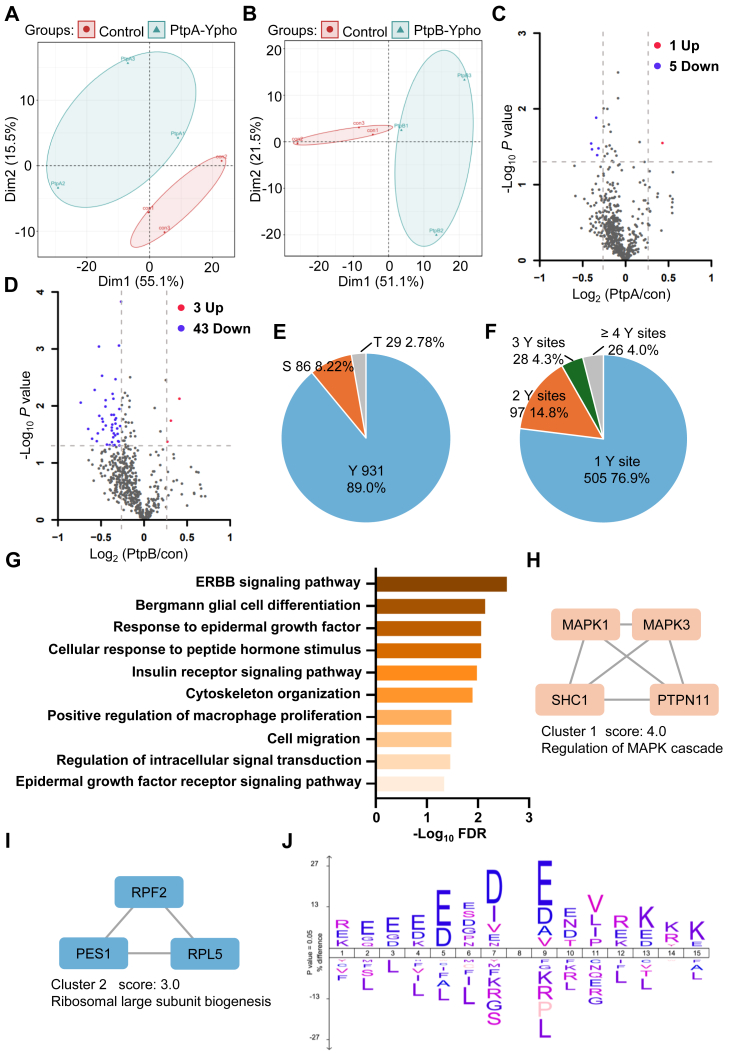
**Global landscape of PtpA/PtpB affected host phosphoproteome.***A* and *B*, phosphoproteomic principal component analysis (PCA) of (*A*) PtpA or (*B*) PtpB overexpression groups compared to the control. *C* and *D*, Volcano plot of tyrosine phosphoproteome quantification after overexpressed with (*C*) PtpA or (*D*) PtpB. *E*, number and ratio of identified Ser/Thr/Tyr phosphosites. *F*, number and ratio of proteins containing 1/2/3/≥ 4 identified tyrosine phosphosites. *G*, gene ontology biological process (GO-BP) enrichment analysis of tyrosine phosphorylation downregulated proteins after expressing PtpB. *H* and *I*, protein-protein interaction (PPI) analysis of tyrosine phosphorylation downregulated proteins of (*H*) cluster 1 with a score of 4.0 and (*I*) cluster 2 with a score of 3.0. *J*, Motif conservation analysis of dephosphorylated tyrosine sites surrounding the sequence after overexpression of PtpB.

Initially, we confirmed that PtpA/PtpB reduced host tyrosine phosphorylation levels through Western blot analysis. The results indicated a reduction in tyrosine phosphorylation signals in the groups overexpressing PtpA/PtpB compared to the empty vector (EV) control group (Supplemental Fig. S1). In the proteomic analysis, no statistically significant changes in protein levels were observed (Supplemental Fig. S2 and Supplemental Table S1), indicating that PtpA/PtpB did not affect host protein expression or degradation of host proteins. Besides,

To investigate how PtpA/PtpB affect host signal transduction, we conducted the tyrosine phosphoproteomics using SH2-CNBR superbinders. Principal component analysis (PCA) effectively differentiated between the PtpA/PtpB overexpression and EV groups in tyrosine phosphoproteomic data (Fig. 1, *A* and *B*). This observation aligns with our expectations, since PtpA and PtpB are tyrosine phosphatases that alter phosphorylation levels and differentiate the phosphoproteome compared to the control. We identified a total of 931 phosphorylation sites with tyrosine phosphorylation enrichment ratio of 89.0% across all phosphorylation sites detected in our dataset (location probability ≥0.75, Fig. 1*E*). Over 23% identified tyrosine proteins contain multiple tyrosine phosphorylation sites (Fig. 1*F*). In the PtpA overexpression group, five tyrosine phosphorylation sites exhibited downregulation, whereas one site demonstrated upregulation (Fig. 1*C*, 1.2-fold change and *p* value < 0.05). Due to the limited changes observed at the sites, no pathways were significantly enriched when analyzed using KEGG or GO-BP database. The results indicated that PtpA's tyrosine phosphatase activity may not significantly impact host tyrosine phosphorylation. In PtpB overexpression group, 43 tyrosine phosphorylation sites were down-regulated and only 3 sites were up-regulated (1.2-fold change and *p* value < 0.05, Fig. 1*D* and Supplemental Table S2), indicating that tyrosine phosphorylation was notably diminished by PtpB in the host.

Pathway enrichment analysis of downregulated proteins revealed a significant enrichment in pathways associated with signal transduction, including the ERBB signaling pathway, the epidermal growth factor receptor signaling pathway, and macrophage proliferation regulation (FDR <0.05, Fig. 1*G* and Supplemental Table S3). Furthermore, an analysis of protein-protein interactions (PPI) revealed two clusters: cluster 1, associated with MAPK cascade regulation with a score of 4.0 (Fig. 1*H*), and cluster 2, associated with ribosomal biogenesis (Fig. 1*I*). The MAPK cascade represents a crucial receptor tyrosine phosphorylation signal transduction pathway in macrophage immune response and metabolic regulation (25, 26). MAPK1/3, also referred to as ERK1/2, is central in both ERBB and MAPK signaling pathways. Previous studies reported that ERK1/2 would form dimers after T187 and Y189 (T202 and Y204) phosphorylation, and phosphorylated dimers could entry the nucleus into phosphorylate transcription factors and thus regulate gene transcription (25). We conducted an analysis of the sequence motifs surrounding dephosphorylated tyrosine sites in PtpA/PtpB tyrosine phosphoproteomics. Our findings indicated that glutamic acid at −3 and +1 position, as well as aspartic acid at the −1 position were highly conserved (ratio <1, Fig. 1*J* and Supplemental Fig. S3). In conclusion, our study initially identified the potential global substrates of Mtb tyrosine phosphatase in host cells. We found that PtpB significantly affected host tyrosine signaling pathway by reducing protein phosphorylation, which may disrupt the macrophage infection defense and impair the immune response.

### PtpB Decreases ERK1/2 Phosphorylation by Directly Interacting with ERK1/2

**Fig. 2:**
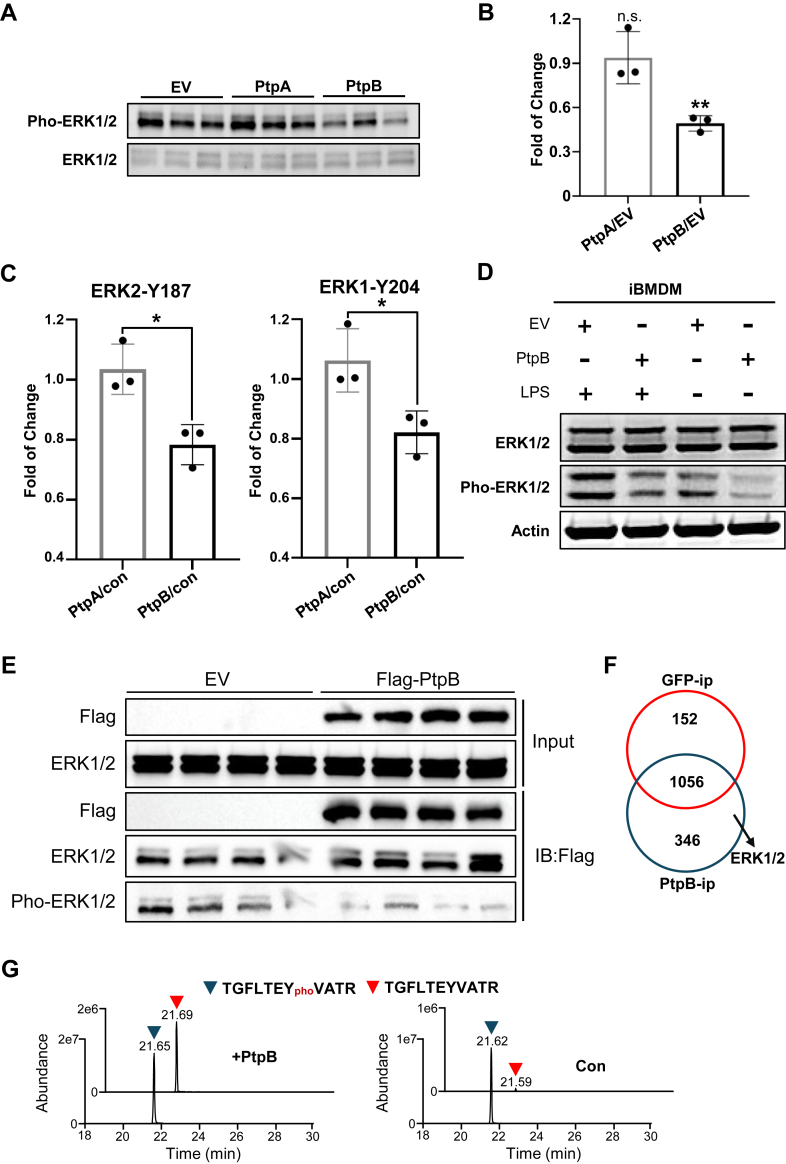
**PtpB decreased ERK1/2 phosphorylation by directly interacting with ERK1/2.***A*, immunoblotting of ERK1/2 protein and phosphorylation after overexpressed with EV, PtpA, and PtpB in HEK-293T cells. *B*, quantitation of phosphorylated ERK1/2 in *A*. *C*, fold change of phosphorylation level in ERK1-Y204 and ERK2-Y187 identified in phosphoproteomic data. *D*, immunoblotting of ERK1/2 protein and phosphorylation in EV or PtpB overexpressed iBMDM cells after stimulated with LPS. *E*, coimmunoprecipitation analysis for PtpB-ERK1/2 interaction. *F*, maxquant software search results for GFP or PtpB coimmunoprecipitation. *G*, peptide dephosphorylation analysis after treated with PtpB.

PtpB was observed to decrease the tyrosine phosphorylation level, primarily affecting the phosphorylation signal transduction pathways, with a particular emphasis on the ERK1/2 signaling pathway. To elucidate the potential effects of PtpB on signal transduction and immune response, we focused on ERK1/2 signaling pathway. Initially, we confirmed that PtpB decreases ERK1/2 phosphorylation through Western blot analysis (Fig. 2*A*). The results showed that PtpB decreased ERK1/2 phosphorylation by approximately 50% compared to the EV control (Fig. 2*B*). Furthermore, phosphoproteomic analysis demonstrated a substantial reduction in phosphorylation at the ERK1-Y204 and ERK2-Y187 sites (ratio <0.83, *p* < 0.05) after PtpB overexpression (Fig. 2*C*). Furthermore, we observed a reduction in ERK1/2 phosphorylation levels following the overexpression of PtpB in the iBMDM cell line (Fig. 2*D*). This finding was consistent with results obtained from PtpB overexpression in the HEK-293T cell line, thereby corroborating that PtpB reduces ERK1/2 phosphorylation in both macrophages and other cell types (Fig. 2, *A* and *D*). To determine if PtpB directly decreases ERK1/2 phosphorylation levels, we initially conducted co-immunoprecipitation (CoIP) assays. The results showed that ERK1/2 were enriched in the PtpB overexpression group, whereas the control group exhibited lighter gray bands, indicating that PtpB could interact directly with ERK1/2 (Fig. 2*E*). We further identified the CoIP proteins using LC-MS/MS, and the data showed 346 proteins were unique to the PtpB group, and ERK1/2 was exclusively identified within this group (Fig. 2*F* and Supplemental Table S4). To verify that PtpB dephosphorylates ERK1/2, we artificially synthesized the phosphorylated peptide corresponding to the amino acid sequences 198 to 208/181 to 191 of ERK1/2. Following the incubation of the peptide with PtpB in the reaction buffer, the presence of the dephosphorylated peptide was observed. In contrast, no obvious dephosphorylation peptide was detected in the GFP control group (Fig. 2*G*). Besides, we also measured ERK1/2 phosphorylation levels after IP with PtpB and found that ERK1/2 phosphorylation levels decreased remarkably, which also verifies that PtpB dephosphorylates ERK1/2 (Fig. 2*E*).

Consequently, our investigation revealed that Mtb phosphatase PtpB decreases host protein ERK1/2 phosphorylation through direct interaction and then confirmed PtpB’s activity using an *in vitro* peptide dephosphorylation assay. This finding advances our comprehension of the mechanisms by which bacterial functional proteins reprogram host signal transduction through post-translational modification.

### PtpB Inhibits ERK1/2 Nuclear Translocation and Suppresses TNF and IL-1β Cytokines Expression

**Fig. 3:**
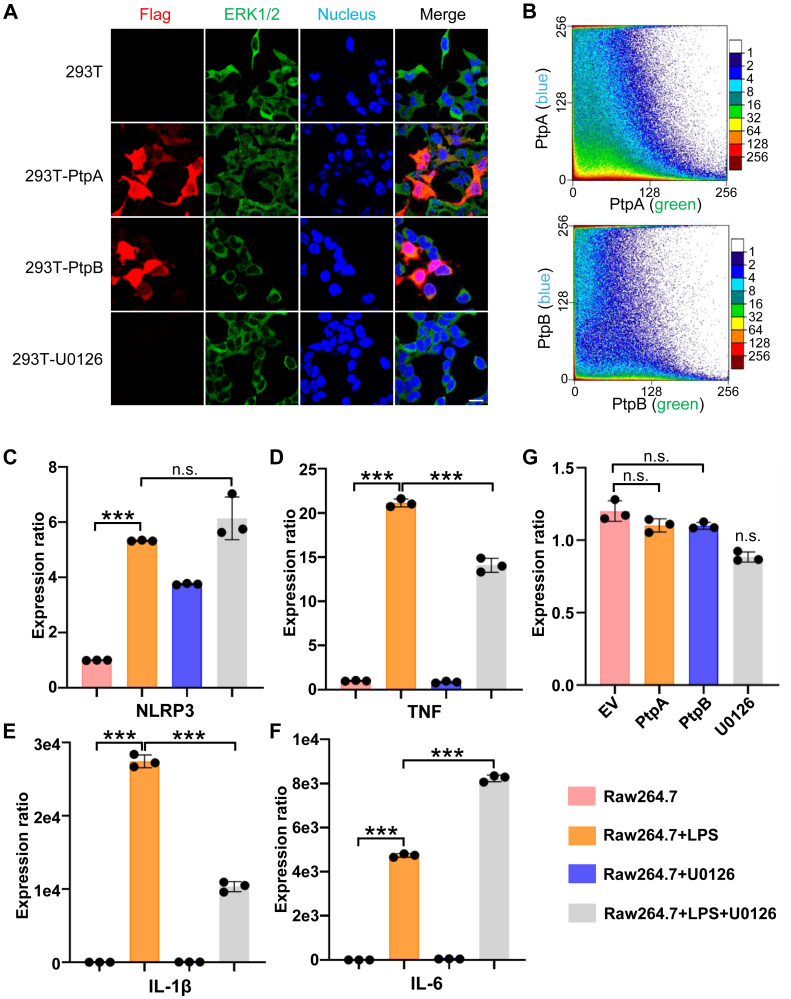
**PtpB inhibits ERK1/2 nuclear translocation and suppresses TNF and IL-1β cytokine expression.***A*, subcellular localization of ERK1/2 after overexpressing PtpA/PtpB or treating with U0126. Scale bar, 20 μm. *B*, density scatter plot analysis of ERK1/2 and nucleus after overexpressed with PtpA or PtpB. *C*–*F*, cytokine transcription levels after being treated with U0126 or LPS in Raw264.7 cells. *G*, the NF-κB promoter transcriptional activity measurement after expressing PtpA, PtpB, or treating with U0126 in HEK-293T cells. All the data are presented as means ± SEM, and ∗*p* < 0.05, ∗∗*p* < 0.01, ∗∗∗*p* < 0.001. Two-tailed unpaired Student’s *t* test was used for statistical analysis.

ERK1/2 are pivotal proteins within the MAPK (Mitogen-Activated Protein Kinase) signaling pathway. Upon recognition of extracellular stimulation by receptor tyrosine kinases (RTKs), a series kinase could be activated and resulted in ERK1/2 phosphorylation (25, 26). Phosphorylated ERK1/2 typically form dimers and then translate to the nucleus. Activated ERK1/2 dimers enhance transcription of target genes and it’s important for regulating gene expression (27). To verify the impact of PtpB on ERK1/2 activation and nuclear translocation, we overexpressed PtpB in HEK-293T cells. Immunofluorescence (IF) analysis demonstrated that most ERK1/2 proteins were located in the cytoplasmic region rather than the nucleus (Fig. 3*A*). Compared to the group with PtpA overexpression, PtpB significantly reduced the nuclear level of ERK1/2. As shown in Figure 3*B*, PtpB overexpression inhibited the colocalization of ERK1/2 with the nucleus, whereas PtpA did not exhibit significant changes in colocalization, as determined by density dot analysis. Additionally, to verify whether cytokine expression depends on the ERK pathway, we employed ERK-specific inhibitors to assess the functional impact of ERK phosphorylation modulation on cytokine production. We evaluated the inhibitory effects of ERK1/2 phosphorylation inhibitor, specifically U0126 (28) and SCH772984 (29, 30), and determined that U0126 demonstrated superior efficacy (Supplemental Fig. S4*A*). In SCH772984-treated samples, MDP stimulation continued to enhance ERK1/2 phosphorylation levels, but the increase degree was less pronounced compared to the control. Phosphorylation level was diminished in the Raw264.7 cell line upon treatment with U0126, irrespective of MDP stimulation (Supplemental Fig. S4*A*). We additionally confirmed that lipopolysaccharide (LPS) activates ERK1/2, and this activation was inhibited by U0126 (Supplemental Fig. S4*B*).

ERK1/2 phosphorylation exerts a significant influence on cell proliferation, metabolism and various signaling pathways, including cytokine expression. To investigate the impact of ERK1/2 phosphorylation on cytokine-mediated immune responses, we used LPS-stimulated Raw264.7 cells as a positive control and U0126-treated cells as a negative control. We first determined that ERK1/2 phosphorylation does not affect NLRP3 signaling pathway, indicating that dephosphorylation of ERK1/2 by PtpB was not associated with cell pyroptosis (Fig. 3*C*). Subsequently, we quantified various cytokines to investigate the correlation between ERK1/2 phosphorylation inhibition and cytokine expression. TNF and IL-1β expression levels were upregulated when stimulated with LPS, and downregulated after treating with U0126, which indicated that ERK1/2 phosphorylation could regulate these cytokine expression (Fig. 3D and 3E). Additionally, the expression levels of IL-12b and IL-18 were elevated after treating with U0126 or LPS plus U0126, indicating that U0126 might activate these cytokines *via* alternative signaling pathways (Supplemental Fig. S4*C*). Liu *et al.* elucidated that the cytokine downregulation is associated with PtpB phosphoinositide phosphatase activity rather than tyrosine phosphatase activity (4). However, they did not provide a detailed explanation of the mechanisms by which PtpB influences the expression of TNF and IL-6 cytokines. Regrettably, no downregulation of IL-6 was observed following treatment with U0126, as compared to samples stimulated with LPS (Fig. 3*F*). We also excluded the possibility that U0126 influenced the phosphorylation of other classical proteins, such as NF-κB, which could downregulate the expression of these cytokines. No notable alterations in NF-κB promoter transcriptional activity were detected following treatment with U0126 or PtpB (Fig. 3*G*). We confirmed that Mtb protein PtpB could inhibit the nuclear translocation of ERK1/2. Additionally, LPS-induced levels of TNF and IL-1β were reduced by the ERK1/2 phosphorylation inhibitor U0126. Our findings expand the potential pathway by which ptpB regulates the expression of IL-1β, and indicate that PtpB inhibits ERK1/2 nuclear translocation and suppresses cytokine expression, providing significant insights into the role of PtpB in the interaction between Mtb and the host.

### PtpB Could Downregulate STAT3 Phosphorylation and affected IL6, IL-1β Expression by JAK1/STAT3 Signal Pathway

**Fig. 4:**
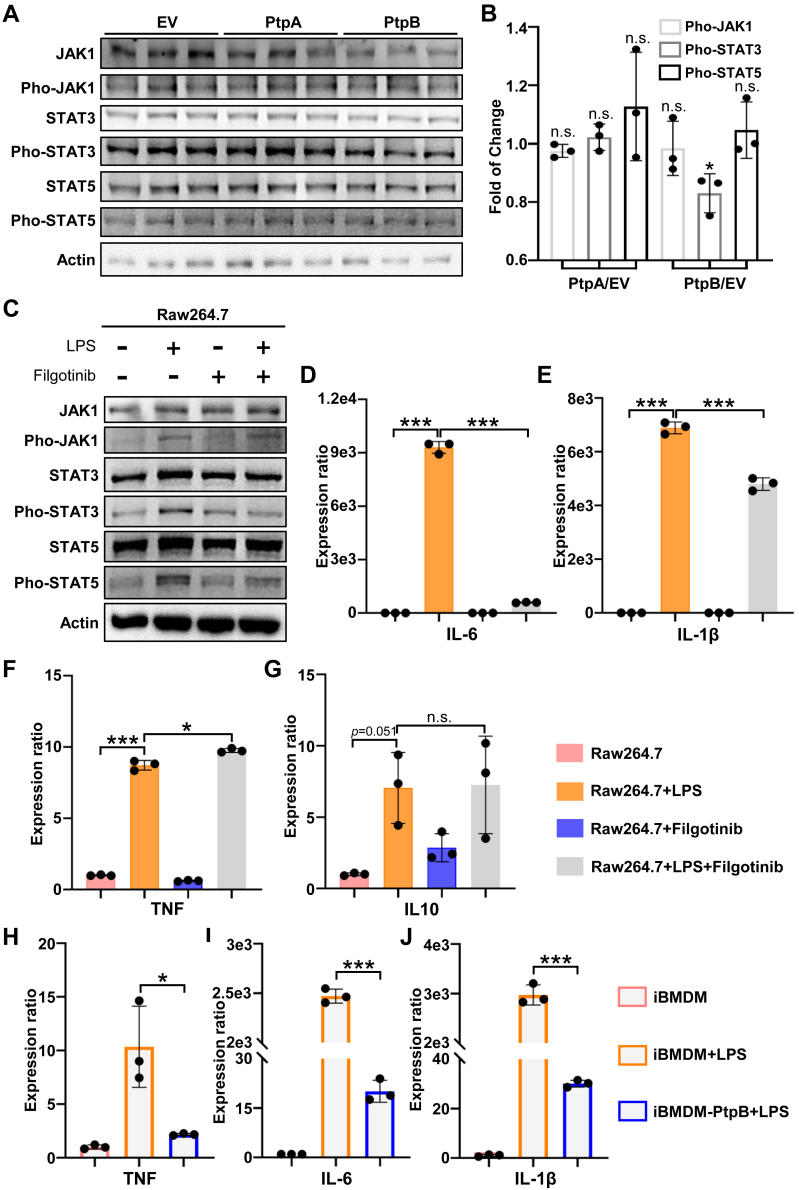
**PtpB inhibits IL-6 expression by reducing STAT3 phosphorylation level**. *A*, proteins and phosphorylated JAK1, STAT3, and STAT5 were examined by western blotting after transfected PtpA or PtpB in HEK-293T cells. *B*, quantitation of phosphorylated JAK1, STAT3, and STAT5 in *A*. *C*, proteins and phosphorylated JAK1, STAT3, and STAT5 were examined by western blotting after being treated with LPS or Filgotinib in Raw264.7 cells. *D*–*G*, cytokine transcription levels after treatment with LPS or Filfotinib in Raw264.7 cells. Macrophages were treated with 5 μg/ml Filgotinib for 1 h and then stimulated with LPS for another 6 h. *H*–*J*, cytokine transcription levels after treatment with LPS in iBMDM and iBMDM-PtpB cell lines. All the data are presented as means ± SEM, and ∗*p* < 0.05, ∗∗*p* < 0.01, ∗∗∗*p* < 0.001. Two-tailed unpaired Student’s *t* test was used for statistical analysis.

Our research indicated that PtpB could reduce ERK1/2 phosphorylation levels, thereby influencing the expression of TNF and IL-1β cytokines. However, the expression of IL-6 cytokine appears to be independent of ERK1/2 regulation. Previous studies indicated that PtpB modulated IL-6 expression *via* mechanisms independent of its phosphoinositide phosphatase activity (4). In our study, we examined JAK-STAT pathway activity during PtpB overexpression, and our results showed that PtpB effectively reduced STAT3 phosphorylation without inducing significant alterations in protein levels when compared to the EV control (Fig. 4*A*). Additionally, we observed a reduction in JAK1 protein level after PtpB overexpression, and its phosphorylation level remained unchanged (Fig. 4, *A* and *B*). This result suggested that JAK1 activity might not be remarkably regulated. We additionally quantified the protein expression and phosphorylation level of downstream STAT3 and STAT5 proteins, and observed that STAT3 phosphorylation was reduced after PtpB overexpression (Fig. 4, *A* and *B*). Thus, PtpB might mediate the reduction in STAT3 phosphorylation, thereby decreasing the expression of STAT3-induced cytokines. To elucidate the involvement of the JAK1-STAT3 signaling pathway in IL-6 expression, we treated macrophages with the JAK1 inhibitor filgotinib and the immune activator LPS, and then observed changes in cytokine expression. Filgotinib inhibited the phosphorylation of JAK1, STAT3, and STAT5, and LPS stimulation only enhanced JAK1 phosphorylation but did not increase the phosphorylation levels of STAT3 and STAT5 (Fig. 4*C*). Following treatment with filgotinib, cytokine expression was assessed, which revealed a significant inhibition of IL-6 expression upon LPS stimulation (Fig. 4D). Furthermore, our findings indicated that the JAK-STAT pathway modulates IL-1β cytokine expression, and showed little influence on TNF and IL-10 levels (Fig. 4, *E*–*G*). Furthermore, we expressed PtpB in immune cells to directly assess its regulatory function. As shown in Figure 4, *H*–*J*, iBMDM cells showed significant activation upon LPS treatment; however, cytokine transcription levels were reduced in PtpB-expressing cells, demonstrating PtpB's direct modulation of cytokine expression. In conclusion, our findings suggest that PtpB decreased TNF and IL-6 cytokine expression by reducing ERK1/2 and STAT3 phosphorylation. Furthermore, IL-1β expression was co-regulated by the NLRP3, ERK1/2, and JAK-STAT pathways, relying on the phosphoinositide and tyrosine phosphatase activities of PtpB.

### PtpB Enhances Bacterial Intracellular Survival and Inhibits Cytokine Expression by Decreasing ERK1/2 Activity

**Fig. 5:**
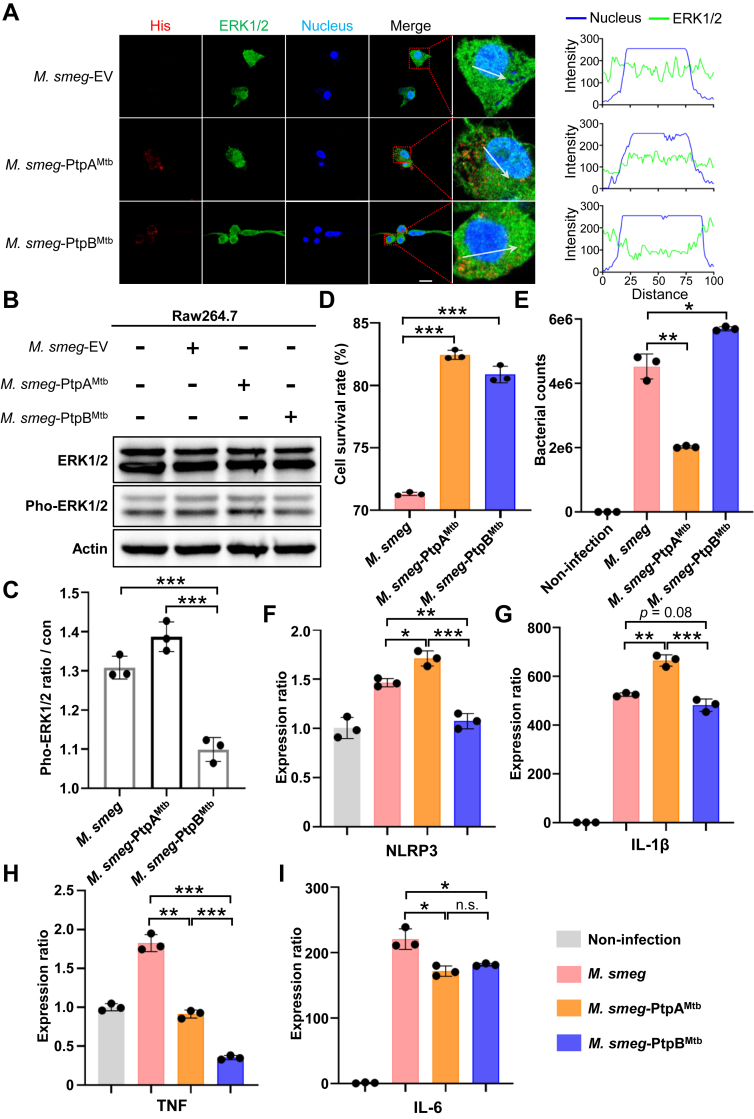
**PtpB promotes bacterial intercellular survival and inhibits cytokine expression during infection**. *A*, *M. smeg expressed with PtpA or PtpB was used to infect Raw264.7 cells for 24 h, and immunofluorescence showed the subcellular location of ERK1/2*. Scale bar, 20 μm. *B*, ERK1/2 protein and phosphorylation were measured by western blotting in Raw264.7 cells after being infected by *M. smeg*-PtpA^Mtb^ or *M. smeg*-PtpB^Mtb^. *C*, quantitation of phosphorylated ERK1/2 in *A*. *D* and *E*, macrophages' survival rate and (*E*) bacterial counts were measured after being infected with *M. smeg*, *M. smeg*-PtpA^Mtb,^ or *M. smeg*-PtpB^Mtb^. *F*–*I*, cytokine transcription levels in macrophages after being infected with *M. smeg*, *M. smeg*-PtpA^Mtb^ or *M. smeg*-PtpB^Mtb^. All the data are presented as means ± SEM, and ∗*p* < 0.05, ∗∗*p* < 0.01, ∗∗∗*p* < 0.001. Two-tailed unpaired Student’s *t* test was used for statistical analysis.

We confirmed that overexpression of ptpB in HEK-293T and iBMDM cells led to a reduction in ERK1/2 phosphorylation. To better mimic native secretion stoichiometry, we constructed *M. smeg* strains with expression of Mtb proteins PtpA or PtpB. We then evaluated the impact of native PtpB’s effect on ERK1/2 dephosphorylation during infection. Raw264.7 cells were infected with *M. smeg*, *M. smeg*-PtpA^Mtb^, or *M. smeg*-PtpB^Mtb^ at a multiplicity of infection (MOI) of 5 for over 24 h. ERK1/2 were predominantly activated during *M. smeg* or *M. smeg*-PtpA^Mtb^ infection, with no significant non-colocalization with the nucleus (Fig. 5*A*). In contrast, *M. smeg*-PtpB^Mtb^ infection resulted in ERK1/2 non-colocalization with the nucleus, suggesting a diminished activation of ERK1/2 (Fig. 5*A*). We additionally analyzed the phosphorylation level of ERK1/2 during infection and observed that the ERK1/2 phosphorylation levels were significantly reduced in the *M. smeg*-PtpB^Mtb^ infected group relative to the other infected groups (Fig. 5*B*). The quantification results showed *M. smeg*-PtpB^Mtb^ infection led to only a 9.9% increase in ERK1/2 phosphorylation levels, whereas the *M. smeg*-PtpA^Mtb^ infected group enhanced around 38.7% (Fig. 5*C*). The macrophage survival rate was higher in the *M. smeg*-PtpB^Mtb^ infected group than in the *M. smeg* group, but lower than in the *M. smeg*-PtpA^Mtb^ group. However, bacterial counts showed that *M. smeg*-PtpB^Mtb^ had the highest survival rate, suggesting that PtpB enhances bacterial intracellular survival and reduces macrophage death (Fig. 5, *D* and *E*). To evaluate cytokine expression during infection, we collected macrophages infected with constructed strains. We measured transcription level of NLRP3 and observed a significant downregulation of gene expression in the group infected with *M. smeg* (Fig. 5*F*). This pathway was not associated with ERK1/2 and has been reported to depend on PtpB-affected phosphatidylinositol phosphorylation signaling pathway (4). Subsequently, we measured the levels of TNF and IL-1β, which were found to be reduced in the *M. smeg*-PtpB^Mtb^ infected group, consistent with the observed inhibition of ERK1/2 phosphorylation (Fig. 5, *G* and *H*). We also found IL-6 cytokine was downregulated, and it’s common with JAK-STAT inhibited results (Fig. 5*I*). Other cytokines were also measured, and the results were consistent with previous reports (Supplemental Fig. S5).

In conclusion, we confirmed that PtpB downregulates TNF, IL-1β and IL-6 by dephosphorylating ERK1/2 during infection. This study elucidated the role of PtpB as a tyrosine phosphatase in the regulation of host cytokines and highlighted the importance of comprehending how Mtb virulence proteins modulate host signaling pathways through diverse mechanisms.

### PtpB is a Significant Virulence Protein in Host Immune Regulating During Mtb Infection

**Fig. 6:**
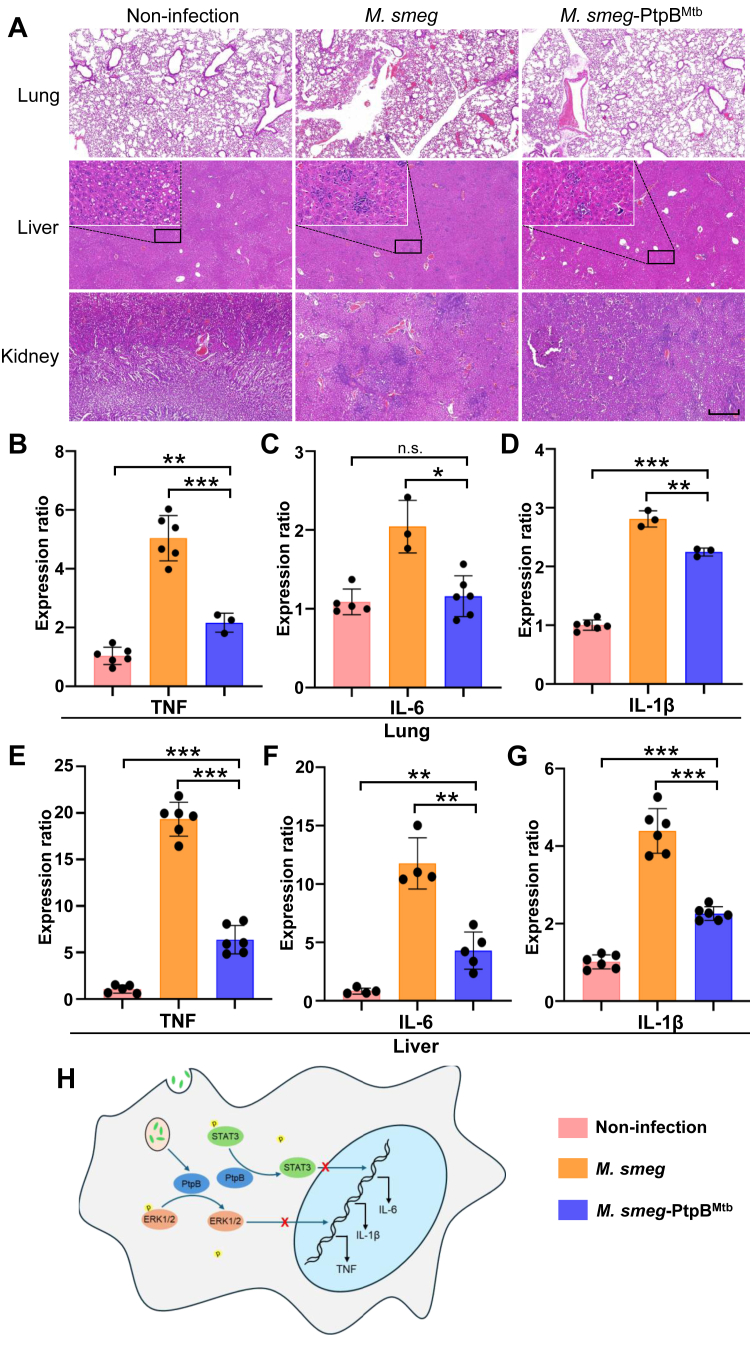
**PtpB could alleviate tissue damage caused by infection.***A*, histopathology of lung, liver, and kidney sections after being infected with *M. smeg* or *M. smeg*-PtpB^Mtb^ for 2 weeks. Boxes indicate sites of cellular infiltration. Scale bar, 0.5 mm. *B*–*G*, cytokine transcription levels in (*B*–*D*) lung and (*E*–*G*) liver after being infected with the indicated strains. All the data are presented as means ± SEM, and ∗*p* < 0.05, ∗∗*p* < 0.01, ∗∗∗*p* < 0.001. Two-tailed unpaired Student’s *t* test was used for statistical analysis. *H*, proposed model for Mtb affects host ERK1/2 and STAT3 phosphorylation and suppresses cytokine expression.

We then explored the function of the PtpB virulence protein during bacterial infection. After infecting mice with *M. smeg* or *M. smeg*-PtpB^Mtb^, we performed hematoxylin and eosin (H&E) staining on tissue samples to examine the pathological alterations. The H&E staining of lung tissue demonstrated alterations indicative of lung injury in the group with *M. smeg* infection (Fig. 6*A*). The histological staining revealed alveolar damage characterized by the disruption of the normal alveolar architecture. Prominent inflammatory of inflammatory cells is observed in the interstitial spaces, and a reduction in symptoms is noted in the *M. smeg*-PtpB^Mtb^ infected group (Fig. 6*A*). Inflammatory infiltration was notably more pronounced in the liver and kidney tissues of the *M. smeg* infected group, whereas a reduction in inflammatory infiltration was observed in the *M. smeg*-PtpB^Mtb^ infected group (Fig. 6*A*). These results indicated that PtpB reduced tissue damage and inflammatory infiltration caused by mycobacterial infection. To further validate the impact of PtpB on cytokine expression in mice, we extracted the RNA from tissue samples and quantified these cytokine transcription levels. The data indicated that the expression levels of TNF, IL6, and IL-1β were significantly reduced in the lungs of the *M. smeg*-PtpB^Mtb^ group compared to the *M. smeg*-infected group (Fig. 6, *B*–*D*), and the similar results were observed in liver (Fig. 6, *E*–*G*). In comparing the cytokine levels between the *M. smeg* and *M. smeg*-PtpB^Mtb^ infected groups, it was observed that certain cytokines exhibited a significant reduction in the *M. smeg*-PtpB^Mtb^ group, whereas others did not show any significant changes. These results were not consistent with our observations at the cellular level (Supplemental Fig. S6). Liu *et al* demonstrated that specific cytokines were reduced *via* the PtpB-PI4P-GSDMD pathway (4). Consistent with these findings, our cellular infection experiments also revealed a significant downregulation of these genes. It is possible that the culture time was insufficient to detect alterations in these cytokines during *M. smeg* and *M. smeg*-PtpB^Mtb^ infections in mice. In conclusion, we confirmed that Mtb virulence protein PtpB could dephosphorylate ERK1/2, thereby impeding its nuclear translocation. This inhibition of ERK1/2 activity led to a marked reduction in the levels of TNF and IL-1β cytokine during the host immune response. Furthermore, we also found that PtpB could dephosphorylate STAT3 and affect IL-6 and IL-1β transcription, which contribute to mycobacterial infection (Fig. 6*H*). Our findings underscored the role of PtpB in bacteria-host interactions and highlighted the capacity of virulence proteins to modulate host immune responses through diverse pathways.

## Discussion

Mtb is an intercellular pathogen that delivers various virulence factors into host cells to facilitate its survival. Numerous researchers have identified several of these virulence factors, such as Pat (Rv0998) (31), UreC (Rv1850) (32), PE_PGRS29 (Rv1468c) (14), SerC (Rv0884c) (33), PstP (Rv0018c) (34). All these factors influence the host immune response through various pathways. Rv0884c is a non-secreting phosphoserine aminotransferase (SerC) which increases Mtb D-serine production under hypoxic conditions. D-serine could interact with WDR24 to inhibit the formation of GATOR2 complex, thereby inactivating the mTORC1-Tbet-IFN-γ pathway in CD8+ T cells (33). However, the majority of virulence factors are enzymes secreted into host cells, where they interact with critical immune response proteins. For instance, Rv1850 has been reported to be secreted into macrophages, where it affects host immune responses and facilitates intercellular survival of Mtb (32). Mtb harbors a large PTM enzyme system, a subset of which may be secreted extracellularly. These enzymes have the potential to disturb signal transduction pathways or interfere with protein binding. Consequently, it’s necessary to explore potential substrates of these virulent factors within the host. Although some articles have identified substrates for these PTM enzymes in both Mtb and the host (12, 35, 36, 37), there remains a notable lack in systematic research focused on tyrosine phosphatase substrates within the host. In this study, we identified the potential substrates of PtpA/PtpB. Our findings indicated that PtpB markedly decreases the phosphorylation levels of proteins involved in the receptor tyrosine signaling pathway, suggesting that PtpB might inhibit host immune responses during infection. The MAPK and JAK-STAT pathways are important in cellular response to environmental stimuli. The phosphorylation events within these pathways disturb the host signal transduction and the immune response. ERK1/2 serve as the terminal proteins in the MAPK pathway and are sequentially activated by Ras and MEK. Phosphorylated ERK1/2 dimerize and translocate to the nucleus, then activate multiple protein translations. The JAK-STAT pathway is essential for mediating immune responses and inflammatory processes. In this pathway, Janus kinases (JAKs) are activated by upstream kinases, which leads to the subsequent phosphorylation of signal transducers and activation of Transcription (STAT) proteins. Signal Transducer and Activator of Transcription 3 (STAT3) is a key regulator in the JAK-STAT pathway. Phosphorylated STAT3 forms dimers and enters the nucleus to activate cytokines and immune-related genes transcription (38, 39). Our study indicated that PtpB possessed the capability to dephosphorylate ERK1/2 and then inhibited their nuclear translocation. In addition, inhibition of ERK1/2 and STAT3 could decrease TNF, IL-6, and IL-1β cytokine expression and reduce immune response.

In conclusion, we conducted a comprehensive global identification of the potential substrates of Mtb tyrosine phosphatases PtpA and PtpB within the host. Our study showed that PtpB could reduce ERK1/2 phosphorylation levels, inhibit its nuclear translocation and suppressing TNF and IL-1β cytokines expression. Additionally, PtpB could lead to a decrease in STAT3 phosphorylation level to inhibit JAK-STAT signaling pathway, which reduced IL-6 and IL-1β cytokines expression (Fig. 6*H*). Our research highlighted the tyrosine phosphatase activity of PtpB during infection and its role in affecting host immune response. These findings might identify a prospective therapeutic target for future anti-TB therapies.

## Data Availability

All mass spectrometry raw data have been deposited to the iProX Consortium with Project ID: IPX0009812001 (https://www.iprox.cn/page/SSV024.html;url=1755861357314t7vI. Password: iORs).

## Supplemental data

This article contains supplemental data (4, 40).

## Conflict of Interest

The authors declare that they do not have any conflicts of interest with the content of this article.
