# Supplementary material for: Mycobacterial Tyrosine Phosphatase PtpB Affects Host Cytokine Expression by Dephosphorylating ERK1/2 and STAT3
Source: Mol Cell Proteomics. 2025 Sep 9;24(10):101067. doi: 10.1016/j.mcpro.2025.101067 (PMC12546780; doi:10.1016/j.mcpro.2025.101067)
Supplement: Supporting information [file mmc7.docx]

**Supporting Information**

**Mycobacterial tyrosine phosphatase PtpB affects host cytokine expression by dephosphorylating ERK1/2 and STAT3**

Tianxian Liu,^1,2^ Yameng Fan,^2^ Yijia Chen,^1^ Shuyu Xie,^2^ Jun-Yu Xu,^2,3,*^ Minjia Tan,^2,3,*^ Bang-Ce Ye,^1,*^

^1^ Laboratory of Biosystems and Microanalysis, State Key Laboratory of Bioreactor Engineering, East China University of Science and Technology, Shanghai, China

^2^ State Key Laboratory of Drug Research, Shanghai Institute of Materia Medica, Chinese Academy of Sciences, Shanghai, 201203, China

^3^ Zhongshan Institute for Drug Discovery, Shanghai Institute of Materia Medica, Chinese Academy of Sciences, Zhongshan 528400, China

* Corresponding author: Bang-Ce Ye (bcye@ecust.edu.cn); Minjia Tan (mjtan@simm.ac.cn); Jun-Yu Xu (jyxu@simm.ac.cn).


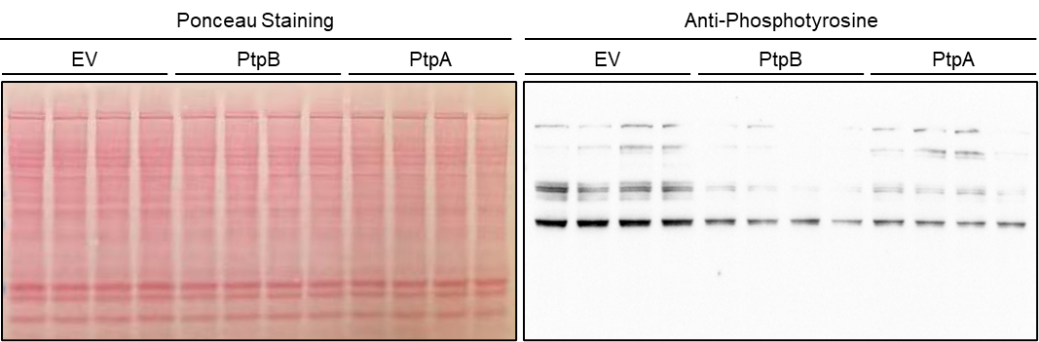


Figure S1. Western blotting analysis of global tyrosine phosphorylation level by antiphosphotyrosine antibody in HEK-293T cells. Samples were transfected with EV, PtpA, or PtpB.


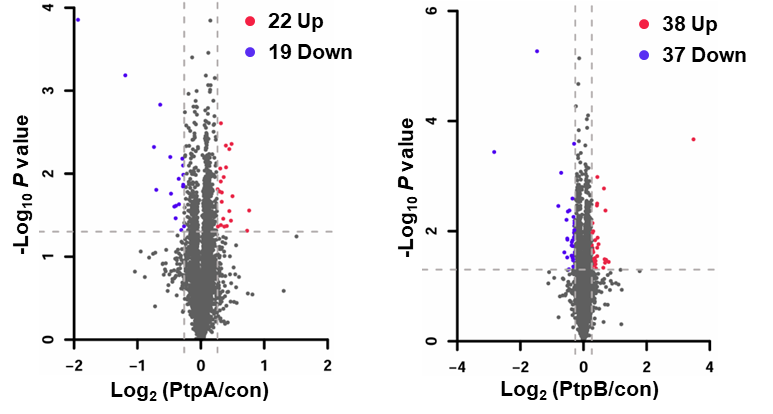


Figure S2. Volcano plot of proteome quantification after expressed with PtpA or PtpB in HEK-293T cells.


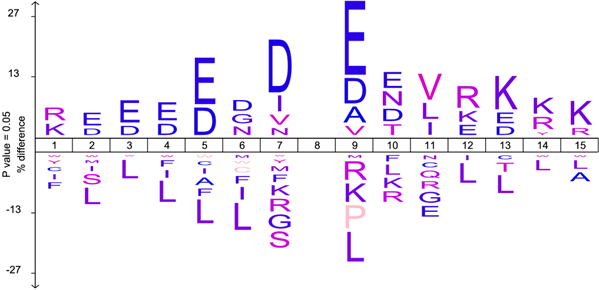


Figure S3. Motif conservation analysis of dephosphorylated tyrosine sites surrounding sequence after overexpressed PtpA.


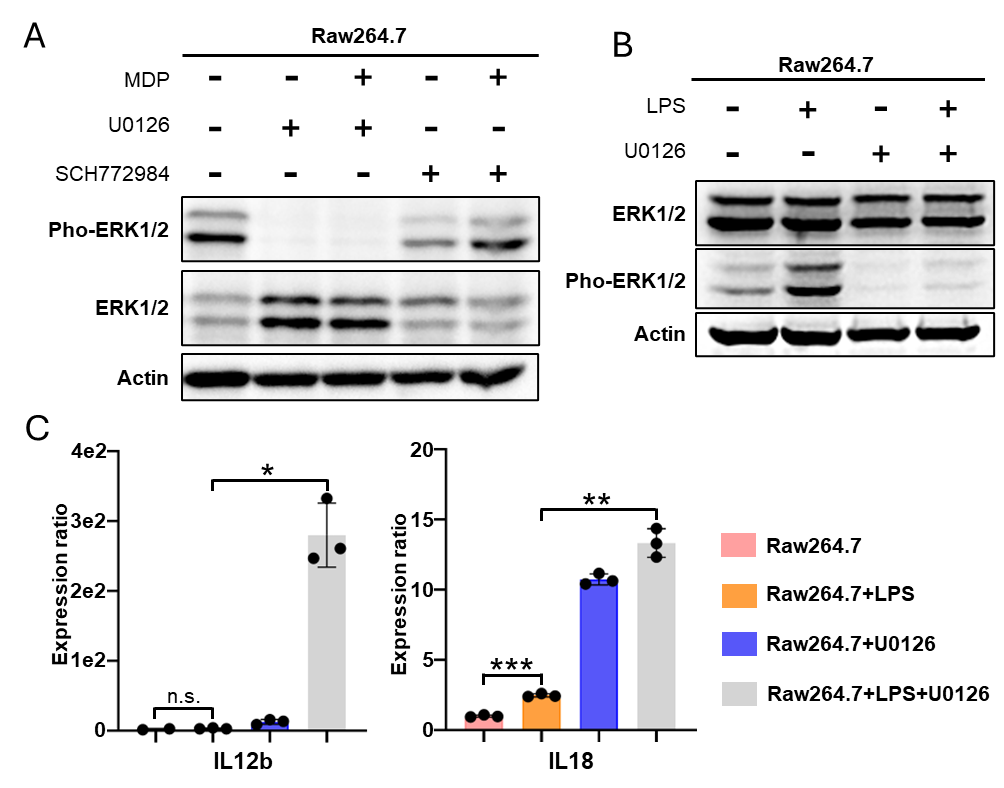


Figure S4. ERK phosphorylation and cytokine transcription levels after treated with U0126 or LPS in Raw264.7 cells. **(A and B)** Immunoblotting protein and phosphorylation of ERK1/2. (A) Samples were treated with 10 μg/mL U0126 or SCH772984 for 4 hours and MDP for another 12 hours or (B) LPS for another 6 hours. **(C)** Cytokine transcription levels after treated with U0126 or LPS in Raw264.7 cells.


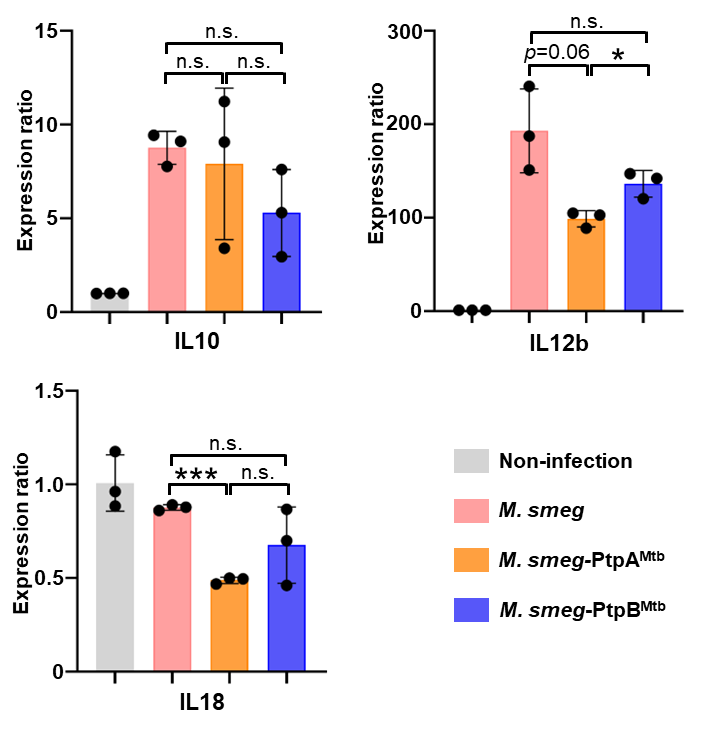


Figure S5. Cytokines transcription levels in macrophages after infected with *M. smeg*, *M. smeg*-PtpA^Mtb^ or *M. smeg*-PtpB^Mtb^.


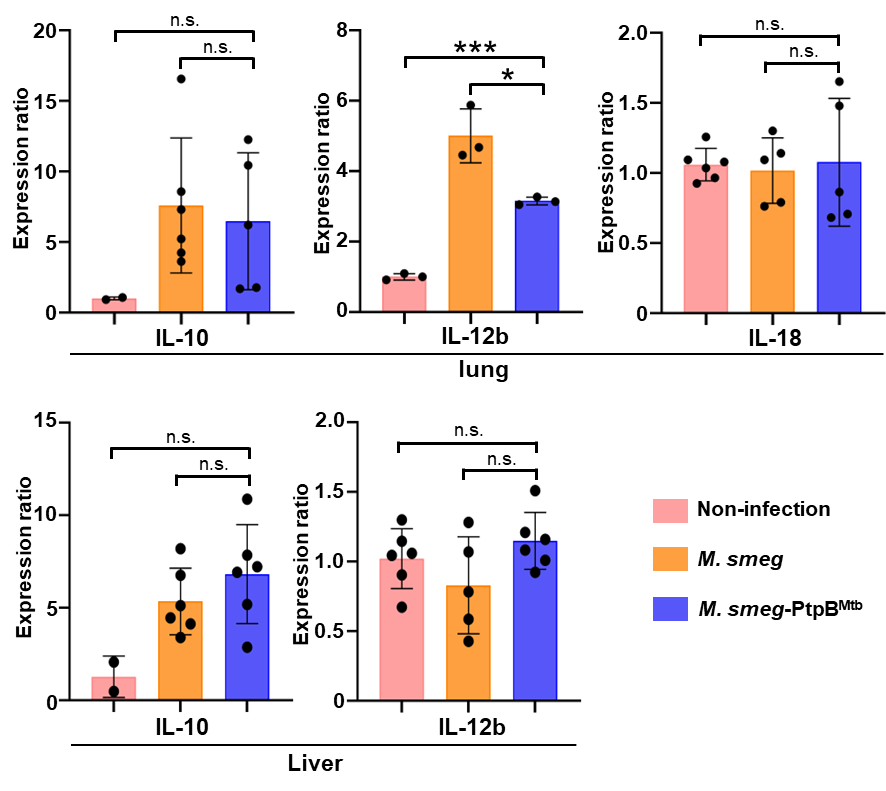


Figure S6. Cytokines transcription levels in lung and liver after infected with *M. smeg* or *M. smeg*-PtpB^Mtb^.
